# CAR Intrinsic Design Pre-Shapes Transcriptional and Metabolic Networks in CAR T Cells

**DOI:** 10.3390/metabo16010052

**Published:** 2026-01-07

**Authors:** Didem Agac Cobanoglu, Samantha Franklin, Yue Hu, Devon J. Boland, Xiaotong Song

**Affiliations:** 1 Department of Translational Medical Sciences, School of Medicine, Texas A&M University, Houston, TX 77030, USA; 2 Center for Infectious and Inflammatory Diseases, Institute of Biosciences and Technology, Texas A&M University, Houston, TX 77030, USA; 3 Texas A&M Institute of Genome Sciences & Society (TIGSS), Texas A&M University, College Station, TX 77845, USA; srfranklin@tamu.edu (S.F.); devonjboland@tamu.edu (D.J.B.)

**Keywords:** chimeric antigen receptors, NF-kB, transcription, RNA-seq

## Abstract

Background/Objectives: Chimeric antigen receptor (CAR) T cells are a powerful cancer therapy, but their function depends heavily on internal signaling domains and metabolic adaptability. Most studies evaluate CAR behavior upon antigen exposure, yet intrinsic signaling properties may pre-program CAR T cell states even in the absence of stimulation. This study investigates how CAR design and metabolic support shape baseline transcriptional programs, focusing on tonic signaling and NF-κB-related pathways. Methods: We engineered CAR T cells targeting HER2 or GPC3 antigens, incorporating either 4-1BB or CD28 co-stimulatory domains, respectively. A subset of cells was further modified with adenosine deaminase 1 (ADA1) and CD26 to degrade extracellular adenosine and supply inosine, a metabolic strategy termed metabolic refueling (MR). Bulk RNA-seq was performed on resting T cells without antigen stimulation. We analyzed differential gene expression, gene set enrichment (GO, KEGG, Hallmarks), and transcription factor activity (DoRothEA) to assess the impact of CAR design and MR on T cell programming. Results: All CAR T cells exhibited activation of NF-κB–centered inflammatory programs at baseline, indicating tonic signaling. GPC3 CAR T cells showed stronger baseline activation than HER2 CAR T cells. Metabolic refueling amplified these programs without altering their directionality, enhancing inflammatory, survival, and effector modules. Transcription factor activity scores mirrored these trends, highlighting RELA, FOS, and STATs as key regulatory nodes. Conclusions: CAR-intrinsic features, notably co-stimulatory domain choice, define the tonic NF-κB activation tone in resting CAR T cells. Metabolic refueling boosts these baseline states without overstimulation, suggesting it may be especially valuable for weaker CAR constructs. These findings provide a framework for tuning CAR T cell function through combinatorial design strategies targeting signaling and metabolism.

## 1. Introduction

Engineered chimeric antigen receptors (CARs) couple antigen recognition to intra-cellular costimulatory programs, enabling T cells to activate with a speed and amplitude that exceed native TCR responses. This supraphysiologic activation is essential for tumor clearance but can be difficult to sustain in solid tumors, where antigen density varies spatially, metabolites such as adenosine blunt receptor signaling [1], and chronic stimulation drives differentiation trajectories toward dysfunction [2].

Glypican-3 (GPC3) and Human Epidermal Growth Factor 2 (HER2) represent two clinically relevant solid-tumor targets with distinct biology. GPC3 is overexpressed in hepatocellular carcinoma, often within inflamed, hypoxic microenvironments [3]; HER2 is expressed across diverse epithelial cancers with variable surface density [4]. We have previously shown that metabolic refueling (MR) of CAR T cells via ADA1/CD26 improves anti-tumor responses. MR cells can degrade extracellular adenosine and supply inosine as an alternative carbon source [5]. By lowering adenosine, MR reduces signaling through adenosine alpha 2A and 2B receptors (A2AR/A2BR) that would otherwise dampen cAMP-PKA–linked T-cell activation. At the same time, inosine can enter central carbon metabolism [6], supporting bioenergetic demands during repeated antigen engagement.

NF-κB-driven transcriptional programs sit at the nexus of several functional pathways: NF-κB shapes cytokine and chemokine secretion, survival, metabolic set points, and fate decisions [7]. NF-κB “tone” is not simply good or bad: insufficient activity compromises persistence and effector function, whereas excessive or tonic activity can precipitate apoptosis, exhaustion, or inflammatory toxicity [8]. Tuning NF-κB to a productive intermediate range, therefore, becomes a central design goal for next-generation CAR T cells. Indeed, genetic evidence shows that the canonical NF-κB pathway is crucial for T cell survival and memory maintenance, while continuous NF-κB activation (for instance, due to tonic CAR signaling) can drive T cells into apoptosis or dysfunction [9]. In other words, optimal CAR T cell function likely requires an NF-κB balance that avoids both signal insufficiency and overload [10].

Since NF-κB integrates costimulatory signaling, survival programs, cytokine output, and metabolic state, baseline NF-κB–associated transcriptional tone may reflect how CAR intrinsic design pre-conditions T cell state even before antigen exposure. We therefore hypothesized that (i) CAR design features are associated with distinct baseline transcriptional programs detectable in resting CAR T cells in the absence of antigen stimulation, and (ii) metabolic refueling (ADA1/CD26) amplifies or stabilizes these baseline activation-associated programs without necessarily introducing qualitatively distinct pathway directionality. We hypothesize that MR allows NF-κB modules to be engaged robustly in hostile microenvironments.

We hypothesized that differences in CAR intrinsic signaling–particularly the choice of co-stimulatory domain–and metabolic refueling strategies could be leveraged to achieve this balance. The two CAR constructs used here target GPC3 and HER2 solid tumor antigens but, importantly, they also incorporate distinct co-stimulatory domains (CD28 versus 4-1BB, respectively). Co-stimulatory domain choice is known to influence CAR T cell metabolism, phenotype, and durability [11,12,13,14]. CD28-based CARs tend to drive more glycolytic metabolism and acute effector differentiation, which can yield rapid tumor killing but also faster T cell terminal differentiation and exhaustion [11,15]. By contrast, 4-1BB co-stimulation promotes oxidative metabolism, preserves mitochondrial fitness, and favors a central-memory phenotype associated with improved T cell persistence [11,16,17,18]. Indeed, prior studies have shown that CD28-costimulated CAR T cells often exhibit more pronounced tonic signaling in the absence of antigen—which can enhance early effector functions but also risk premature exhaustion—whereas 4-1BB domains can mitigate tonic signaling and foster a less exhausted state [19]. Consistent with this, CD28-based CAR T cells generally show strong initial NF-κB and PI3K/AKT activation that can wane with continuous stimulation, whereas 4-1BB CAR T cells activate additional non-canonical NF-κB via TRAFs, sup-porting better survival under chronic exposure [11,18]. Thus, comparing a GPC3 CAR (with CD28 signaling domain) to a HER2 CAR (with 4-1BB signaling domain) provides an opportunity to examine how CAR-intrinsic tonic signaling influences baseline T cell transcriptional programs.

In addition, we and others have explored metabolic refueling (MR) as a strategy to bolster CAR T cells in suppressive environments [5]. Metabolic refueling in this context refers to engineering T cells with a membrane-bound CD26 and a cytosolic adenosine deaminase 1 (ADA1) transgene. CD26 serves as a surface anchor for ADA and has intrinsic costimulatory properties, while ADA1 converts extracellular adenosine (a potent immunosuppressive metabolite) into inosine. We have previously shown that this ADA/CD26 metabolic refueling approach improves CAR T anti-tumor activity in solid tumor models. Mechanistically, ADA-expressing CAR T cells can degrade extracellular adenosine—relieving adenosine-A2A receptor signaling that raises intracellular cAMP and inhibits T cell receptor/NF-κB pathways, while the inosine byproduct provides an alternative fuel to support T cell bioenergetics during repetitive stimulation [1]. By lowering adenosine levels and thus reducing chronic A2A receptor engagement on T cells, MR is expected to prevent the PKA-mediated suppression of NF-κB and IL-2 that adenosine normally causes [20]. At the same time, MR CAR T cells should have greater metabolic flexibility (via inosine utilization) to sustain effector functions. We hypothesized that tonic NF-κB activity in CAR T cells could thereby be modulated both by CAR design (CD28 vs. 4-1BB domains) and by metabolic refueling, in the absence of any acute antigen stimulation. Notably, all RNA-seq profiling in this study was performed on resting CAR T cells without antigen exposure, isolating the contributions of CAR-intrinsic signaling in baseline conditions.

To elucidate early transcriptional changes, in the absence of antigenic stimulation, we performed bulk RNA-sequencing from five groups: non-transduced (NT) T cells, GPC3 CAR T, HER2 CAR T, GPC3 MR CAR T, and HER2 MR CAR T cells. We analyzed differential gene expression and applied multiple complementary approaches to interpret pathway-level and regulatory network changes. Gene set enrichment analysis (GSEA) using the MSigDB Hallmark collection was employed to capture broad inflammatory, interferon, and metabolic programs. We also performed enrichment analysis on Gene Ontology (GO) Biological Processes and KEGG pathways to resolve specific biological processes and signaling modules. Because single-gene effect sizes in bulk data can be modest and correlated, we further inferred transcription factor (TF) activities using DoRothEA. These layers, gene sets, pathways, and inferred TF activities provide convergent evidence for which cellular programs are engaged under different CAR designs and MR conditions, rather than relying on any one metric alone.

Our central questions were therefore twofold. First, do CAR T cells with different co-stimulatory domains (CD28 vs. 4-1BB) exhibit distinct baseline signaling tones in the absence of antigen? In other words, does the intrinsic CAR design pre-condition T cells to different transcriptional and signaling states even before encountering tumor antigen? Second, does metabolic refueling (ADA/CD26) shift these baseline states in a manner consistent with improved fitness—for example, elevating productive NF-κB and interferon-stimulated gene activity, or enhancing proliferative and survival pathways—without pushing T cells into an overactivated state? By structuring the analysis to address both axes simultaneously, we aim to identify shared and divergent signatures that can guide improved strategies for CAR T cell design to maximize durability and function in solid tumors.

## 2. Materials and Methods

### 2.1. Data Acquisition

Bulk RNA-seq data analyzed in this manuscript were generated in our prior study (PMID: 38688275) and deposited in the Gene Expression Omnibus (GEO) under accession GSE262447. The dataset contains five experimental groups: non-transduced (NT) T cells, GPC3-CAR T cells, HER2-CAR T cells, GPC3-MR-CAR T cells, and HER2-MR-CAR T cells (n = 3 biological replicates per group). Human peripheral blood mononuclear cells (PBMCs) used for CAR T-cell production were obtained from STEMCELL Technologies (Cat. No. 70025, Vancouver, BC, Canada). The present work constitutes a re-analysis starting from raw FASTQ files using a uniform pipeline to enable consistent read processing, alignment, quantification, and downstream inference across all groups.

### 2.2. Read Quality Control and Trimming

Raw paired-end reads were first assessed for overall quality using FastQC (0.12.1, Babraham Bioinformatics, Cambridge, UK) before and after trimming. Adaptor sequences, low-quality bases (Phred score < 20), and poly-A tails were removed using Trim Galore! (v0.6.10; Babraham Bioinformatics, Cambridge, UK), which wraps Cutadapt for adapter and quality trimming. Poly-A tail removal was applied to reduce mapping artefacts in 3′ regions. Only reads passing quality control after trimming were retained for downstream alignment and quantification.

### 2.3. Alignment and Quantification

Trimmed reads were aligned to the human reference genome (Homo sapiens, GRCh38, Ensembl release 114) using STAR (v2.7.11b, Cold Spring Harbor, NY, USA) [21]. Uniquely aligned reads were then quantified at the gene level using featureCounts (Subread package, v2.0.8, The Walter and Eliza Hall Institute of Medical Research, Parkville, Australia), with the corresponding Ensembl GTF annotation (GRCh38.114) [22]. Only primary alignments from properly paired reads were included in the final gene-level count matrix. The resulting matrix of raw gene counts for all samples was subsequently used as input for downstream differential expression analysis using DESeq2 (v1.42.1) in R (v4.3.1) [23].

The DESeq2 dataset object was created with the reference group set to “NT” (Non-transduced T-cell). Genes were tested for differential expression using the DESeq2 negative binomial generalized linear model framework. Specific contrasts between treatments (i.e., HER2-MR-CAR vs. HER2-CAR, and GPC3-MR-CAR vs. GPC3-CAR) were generated. Principal component analysis (PCA) was performed on variance stabilized data to assess overall sample clustering and potential batch effects. Differentially expressed genes were identified using a relaxed standard of an unadjusted *p*-value < 0.05, and an absolute log2 fold change ≥ 0. Gene symbols were annotated using the org.HS.eg.db database (v.3.18.0). Data visualization and figure generation were performed using ggplot2, EnhancedVolcano, and ComplexHeatmap [24,25,26].

### 2.4. Pathway Analysis

Annotated pathway enrichment analysis was performed using clusterProfiler (v4.10.1) and enrichplot (v1.22.0) R packages. Both Gene Ontology (GO) [27] and Kyoto Encyclopedia of Genes and Genomes (KEGG) [28] databases were queried for enrichment. Significantly enriched terms were defined as those with an adjusted *p*-value 0.05 (BH correction) and a fold enrichment greater than 0.

### 2.5. Transcription Factor Activity Inference

Putative transcription factor (TF) activity was inferred using the human DoRothEA v1 regulon collection, implemented via the decoupleR R package (v.2.8.0) [29]. To address collinearity, TFs with perfect correlation in their target profiles were identified and removed from the interaction network prior to inference. TF activity scores were calculated using decoupleR, incorporating regulons across confidence levels A-D and retaining only those with more than 5 expressed target genes. Resulting TF activity scores were compared between treatment conditions by one-way ANOVA across all samples.

## 3. Results

We have previously shown that metabolic refueling (MR) of CAR T cells via engineered membrane-bound CD26 and cytoplasmic adenosine deaminase 1 improves CAR-T cell therapy efficiency against solid tumors [5]. We analyzed bulk RNA-seq data from resting T cells without antigen exposure across five groups (NT, GPC3-CAR, HER2-CAR, GPC3 MR-CAR, HER2 MR-CAR) from our published dataset (GSE262447) [5]. The CAR products were generated under matched manufacturing conditions and analyzed using a uniform bioinformatics pipeline.

We have first investigated the genes that are differentially expressed between each CAR T cells in comparison to control cells (non-transduced, NT). Differentially expressed genes were identified using adjusted *p*-value <= 0.05 and a log2 fold change of >=0, a relaxed threshold applied to capture a comprehensive expression profile. Compared to NT controls, thousands of differentially expressed genes (DEGs) were found across all CAR products (Figure S1). In the GPC3 model, GPC3 CAR T vs. NT and GPC3 MR CAR T vs. NT shared 5105 genes, corresponding to ~80% and ~86% of their respective DEG sets (totals 6384 and 5954), indicating that MR largely preserves the antigen-driven activation core while adding a modest cohort-specific component (1279 and 849 genes). In the HER2 model, the shared core comprised 1933 genes (~68% of HER2 CAR T vs. NT, 2849 total; ~80% of HER2 MR CAR T vs. NT, 2416 total), leaving 916 and 483 cohort-specific genes, respectively. In other words, CD28 co-stimulatory domain of GPC3 CAR model drove a more extensive transcriptional shift from the baseline (NT) than the 4-1BB co-stimulatory domain containing HER2 constructs.

**Figure 1:**
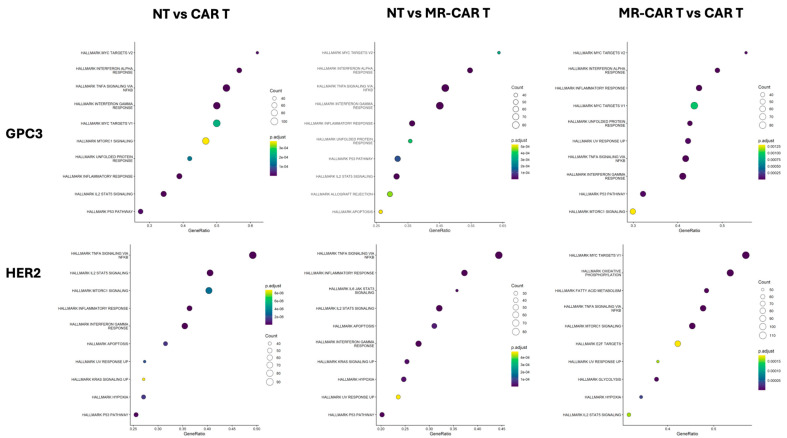
Hallmark pathway enrichment in CAR and MR-CAR T cells reveals tonic activation of NF-κB programs. Gene Set Enrichment Analysis (MSigDB Hallmarks) for NT vs. CAR T, NT vs. MR-CAR T, and MR-CAR T vs. CAR T in GPC3 (top) and HER2 (bottom) are represented. Bubble size indicates the fraction of genes contributing to enrichment; color encodes normalized enrichment score (NES) direction and the adjusted *p* value (FDR). Canonical inflammatory modules—TNF-α via NF-κB, JAK–STAT, and interferon signatures—are enriched in comparison to NT and are further increased in MR-CAR relative to parental CAR, with larger effects in the GPC3 cohort.

We then subjected the DEGs to GSEA against the MsigDB human Hallmark gene sets (Figure 1). Pathways related to inflammation were observed throughout the analysis (e.g., TNF signaling via NF-κB, interferon alpha/gamma signaling, STAT pathways). Both GPC3 CAR T and HER2 CAR T cells are strongly enriched over NT for inflammatory Hallmarks, consistent with activated CAR T cells. This likely reflects tonic CAR signaling—a ligand-independent baseline receptor clustering or signaling—which has been observed especially in some CD28-based CARs and can drive constitutive NF-κB activity [30]. Indeed, our data show that NF-κB-linked pathways were basally elevated in CAR T cells versus NT, consistent with tonic signaling triggering NF-κB–driven genes (Figure 1).

When NT vs. MR CAR comparisons are considered, they showed a similar pattern of pathway enrichment, indicating that the MR constructs maintain activation of key inflammatory programs at levels comparable to standard CAR T cells. When GPC3 and HER2 are compared, GPC3 shows a visible positive shift in inflammatory Hallmarks shown by both a greater number of genes included in the gene sets and higher statistical significance (Figure 1). This shows that GPC3 MR cells had higher pathway engagement with MR, compared to HER2. HER2 shows similar trends in modules, however with much smaller magnitude, showing antigen bias in the activation of transcriptomic modules. In effect, the CD28-based CAR T (GPC3) cells exhibit a “hotter” basal inflammatory profile than the 4-1BB-based CAR T (HER2) cells. This observation aligns with known differences in tonic signaling: CD28 CARs can trigger more spontaneous NF-κB and cytokine signaling in resting T cells, whereas 4-1BB CARs generally require antigen ligation to robustly turn on those pathways, leading to a cooler baseline. Notably, all the major Hallmarks most shifted in our analysis were those tightly coupled to NF-κB and related inflammatory regulators (STATs, interferons, IL-6). Metabolic reprogramming enhanced these programs in both CAR contexts, but more so in the HER2 (4-1BB) case, which started lower.

To formalize these patterns, we quantified the overlap and directionality of Hallmark gene set enrichments. We found that the activation core was large and shared in both CAR types (Figure S2a,b). In the GPC3 model, 24 Hallmark sets were common to GPC3 CAR T vs. NT and GPC3 MR CAR T vs. NT, indicating that MR preserves the activation architecture established by GPC3 CARs. In the HER2 model, the shared core was likewise 24, but 6 and 3 additional sets were unique to HER2 CAR T vs. NT and HER2 MR CAR T vs. NT. Directionality heatmaps (Figure S2c,d) showed that inflammatory modules—TNF-α via NF-κB, IL6–JAK–STAT, and interferon signatures—were positively enriched in both models compared to NT, with concordant directionality between parental CAR and MR-CAR comparisons, indicating that MR programs alter the strength of these pathways rather than inducing divergent pathways. A broad clustering of normalized enrichment scores (Figure S2e) reveals that both CAR and MR-CAR cells show increased inflammatory program activity compared to the NT control with greater effect sizes in GPC3 and detectable but smaller shifts in HER2. Together these data support a model in which NF-κB-centered inflammatory programs define a conserved backbone of CAR T activation, while CAR’s co-stimulatory domain sets the amplitude and MR boosts pathway engagement without changing its directionality.

**Figure 2:**
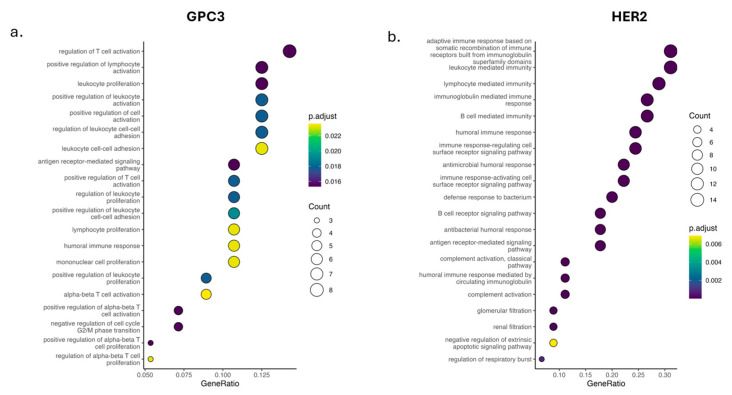
GO Biological Process enrichment for MR-CAR vs. parental CAR within different antigens. (**a**) GPC3 and (**b**) HER2 comparisons. Bubble plots show significantly enriched GO BP terms (adjusted *p* as color; GeneRatio on the x-axis; bubble size = gene count).

We then investigated the gene ontology (GO) Biological Process enrichment for the parental CAR T cells and the MR CAR T cells (Figure 2). Across both antigens, the dominant categories are activation, proliferation, cytokine production, and antigen receptor signaling—processes that canonically sit downstream of NF-κB. Overall trends showed an increase in the enrichment of these pathways in MR CAR T cells compared to the parental CAR T cells; however, the magnitude differs by antigen: incremental amplification on an already high baseline for GPC3 (Figure S1); stronger rescue for HER2 (Figure S2), suggesting that MR can raise effective signaling modules in microenvironments that are otherwise suppressive.

In more detail, GPC3 model (Figure 2a, Figure S3) offers top GO terms in immune activation and effector programs: positive/negative regulation of T-cell activation, lymphocyte proliferation, leukocyte-mediated cytotoxicity, cytokine production, antigen receptor–mediated signaling, and NF-κB–coupled responses. Enrichment of the GO terms is uniformly positive for activation and effector functions, with a balanced representation of proliferation, signaling, and cytotoxicity terms—suggesting that MR enhancing productive activation of CAR T cells. HER2 model (Figure 2b, Figure S4) shows a broader set and higher GeneRatios across activation terms such as adaptive immune response, lymphocyte activation, leukocyte migration/chemotaxis, humoral immune response, and antigen receptor signaling. Compared to GPC3, the larger GeneRatios may suggest in HER2 that MR unmasks or strengthens programs that are weaker at baseline.

These GO enrichments are consistent with increased NF-κB-related genesets: our CAR T constructs depend on CD28 and 4-1BB engagement which relies on the canonical and non-canonical NF-κB. The GO categories enriched (T cell activation, proliferation, cytokine production, etc.) are well-known NF-κB downstream functions in T cells. Together with the Hallmark analysis, Figure 2 supports a model wherein metabolic refueling may boost CAR-intrinsic NF-κB-driven biology across both CAR designs. However, the extent of that boost depends on the initial signaling strength endowed by the CAR’s costimulatory domain: in the CD28 (GPC3) case, MR provides a smaller amplification on an already high baseline, whereas in the 4-1BB (HER2) case, MR provides a larger proportional gain—essentially rescuing the cells from a lower baseline toward a more robust activation state. In both instances, MR may keep NF-κB activity within a productive range, as evidenced by the concurrent induction of NF-κB negative feedback regulators (e.g., TNFAIP3/A20 was upregulated in MR-CAR T cells); that presumably prevent excessive signaling. Together with Hallmark (Figure 1) and the overlap analyses (Figure S2), Figure 2 supports a model in which MR boosts antigen-evoked NF-κB-linked biology, where co-stimulatory domains (CD28 vs. 4-1BB) governing how much of that boost appears as amplification versus rescue. It is important to note that these inferences reflect changes that occur in transcriptomic signatures and do not directly measure signaling dynamics; therefore, we interpret them as pathway-level inference of baseline activation-associated programs.

**Figure 3:**
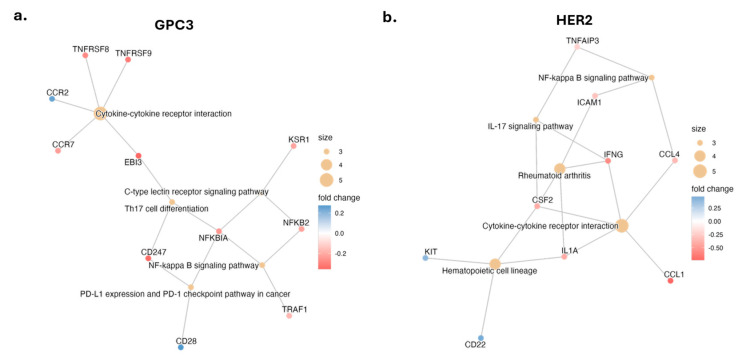
KEGG pathway enrichment for MR CAR T cells vs. parental CAR T cells within different antigens. (**a**) GPC3 and (**b**) HER2 cnet networks derived from KEGG. Pathway nodes are labeled; gene nodes are connected to enriched pathways. Node color encodes log_2_ fold-change (MR CAR T vs. CAR T); node size reflects the number of mapped genes.

When we investigated the KEGG pathways in our dataset (Figure 3, Figure S5), we noticed that across both antigens, KEGG highlights similar pathways: cytokine-cytokine receptor interactions feeding to NF-κB/IL-17 axes with MR increasing pathway engagement. The KEGG network for GPC3 shows cytokine–cytokine receptor interaction as a central hub with connections to NF-κB signaling, C-type lectin receptor signaling, Th17/IL-17 signaling, and PD-L1 expression and PD-1 checkpoint pathway in cancer. This may indicate that CAR T cells upregulate networks of cytokines and receptors that can amplify NF-κB and immune signaling in an autocrine and paracrine fashion. In the GPC3 network, we observed reduced expression of NFKB2 (encoding p100/p52, a non-canonical NF-κB subunit) and TRAF1 in GPC3 MR-CAR vs. GPC3 CAR. These changes could support reduced routing into both canonical and non-canonical NF-κB signaling. We also noted MR-associated decrease in chemokine receptors like CCR2/CCR7 and cytokine subunits like EBI3 (IL-27β), suggesting CAR T cells may better mobilize immune cell trafficking and Th1-polarizing cytokines, suggesting a more inflammatory, effector-ready state.

The KEGG network for HER2 also focuses on cytokine–cytokine receptor interaction, interacting with NF-κB signaling, IL-17 signaling, and additional hematopoietic cell lineage, and adhesion/chemokine nodes (e.g., ICAM1, CCL1/CCL4). Decreases in IFNG and CSF2 suggest a reduction in effector/secretory output in MR CAR T cells. Additionally, the NF-κB pathway node included TNFAIP3 (A20), an inducible negative regulator of NF-κB, which was upregulated in CAR T cells (especially in HER2). A20 expression may be interpreted as a hallmark of robust NF-κB activation that triggers its own feedback loop to prevent overactivation. Given the nature of the transcriptomic data, however, these insights can be interpreted as transcriptional inferences, rather than mechanistic certainties.

**Figure 4:**
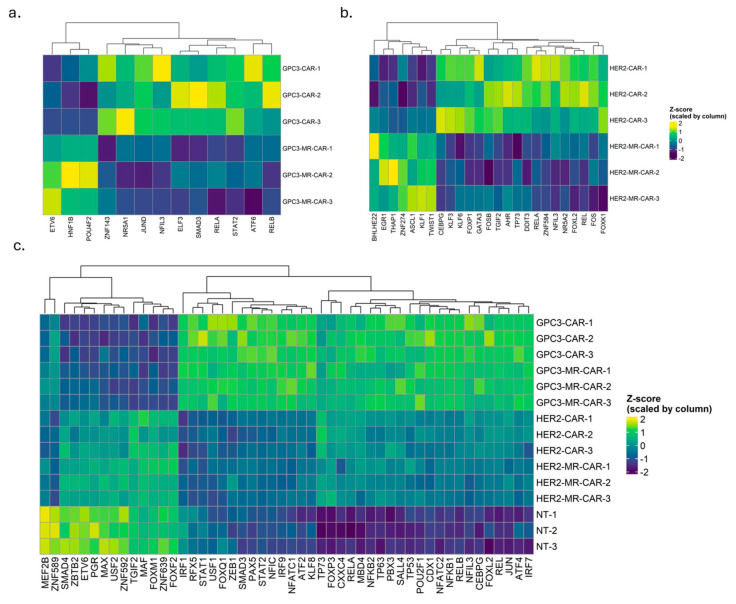
Transcription-factor activity indicates CAR-intrinsic tuning of NF-κB family members. Heatmaps display z-scored TF activities for GPC3 models (**a**), HER2 models (**b**), and across all samples (**c**). Heatmaps display TFs whose activities were found to be statistically significant (*t*-test *p*-value < 0.05, unadjusted).

Finally, we inferred transcription factor (TF) activity from bulk RNA-seq using DoRothEA regulons which provide high-confidence TF-target links aggregated from literature and summarized the inferred activities for each sample (Figure 4). Because DoRothEA activities represent the regulatory influence of a TF on its targets (rather than the TF’s own mRNA abundance), higher activity indicates a target-gene expression pattern consistent with TF engagement.

In all CAR constructs, TFs downstream of inflammatory signaling were prominent. NF-kB family members RELA (p65), RELB and REL, and AP-1 family members FOS, FOSB, JUND showed increased activity in CAR products. This dovetails with our pathway analyses, reinforcing that CAR T cells—even absent antigen—may exhibit a baseline activation of NF-κB– and AP-1–driven transcription. These TFs orchestrate many of the GO processes and cytokines discussed above, underscoring their centrality in CAR T cell function.

Moreover, we assessed the TF activity across all samples, and we noticed that different cohorts of TFs were inferred as active in GPC3 and HER2 constructs. However, when TF activity is assessed between CAR T cells and MR CAR T cells, MR shows limited changes compared to parental construct.

Analysis of TF activity scores across NF-κB-related TFs (RELA/REL), AP-1 (FOS/JUN), and STAT/IRF families reveals only minor fold-change differences between GPC3 MR-CAR T cells and GPC3 CAR T cells, indicating minor modulation of these inflammatory regulators within the GPC3 models (Figure 4a). Similarly, for HER2 CAR T cells, MR introduces only very small absolute changes in TF activity despite sample clustering and Z-score scaling in heatmap representations which create a visually pronounced distinction between MR and parental CAR T groups for both HER2 and GPC3 (Figure 4a,b; Supplementary Table S1). Finally, TF-activity inference supports model-dependent MR effects at the regulatory layer: HER2 shows an MR-associated increase in activities of NF-κB/AP-1/STAT families, whereas GPC3 shows little to modest changes relative to parental CAR. These findings align with the broader transcriptomic view that we present in this paper, where based on transcriptomic data, we suggest that MR may raise transcriptional programming from near-threshold to detectable levels in HER2, while exerting minimal additional gain on the already active GPC3 program.

## 4. Discussion

This study analyzed baseline transcriptional programs in resting CAR and MR-CAR T cells without antigen exposure and identified convergent enrichment of activation-associated inflammatory modules across CAR products. Across all analyses—differential gene expression, gene set enrichment, pathway networks, and inferred TF activities—the data converge on a central theme: CAR T cell basal activation is governed by CAR-intrinsic design, with NF-κB as a tunable hub, and metabolic refueling amplifies this activation without qualitatively altering it. Prior studies have demonstrated that CAR design features, including co-stimulatory domains and CAR expression levels, can influence baseline signaling and tonic signaling propensity [12,13,17,18,31,32,33]. Our data are consistent with this framework, though they do not directly measure signaling dynamics.

In this framework, the CAR’s co-stimulatory domain (and associated tonic signal strength) may establish the baseline tone of NF-κB and related pathways, and metabolic refueling adjusts the amplitude without changing the circuitry’s directionality. This pattern is consistent with MR acting as an amplifier or stabilizer of baseline activation-associated transcriptional programs in resting CAR T cells, rather than inducing a qualitatively distinct transcriptional state. In our prior work, we have shown that ADA1/CD26 engineering improved antitumor activity and function in suppressive environments [5], and inosine has been shown to support T cell bioenergetics under glucose restriction [6].

Our findings have practical implications for CAR T design and therapy optimization. First, they suggest that adding metabolic support (e.g., ADA/CD26 or other strategies to counter immunosuppressive metabolites) may be most beneficial in contexts where the CAR’s intrinsic signaling is on the lower end—such as CARs targeting low-density antigens or utilizing solely 4-1BB co-stimulation. In those cases, MR may lift subthreshold signaling to effective levels, improving T cell activation, inflammatory cytokine output (e.g., IFN-γ, IL-2, GM-CSF), and possibly tissue migration programs (via chemokine receptors). Conversely, in contexts where baseline signaling is already very strong (e.g., a high-avidity antigen with a CD28 CAR), MR serves more as a stabilizer than a primary amplifier. It can maintain function in harsh microenvironments (e.g., by degrading adenosine) without causing additional overactivation—consistent with the observed upregulation of A20 and the lack of a global TF activity spike in the GPC3 model. In such cases, the benefit of MR may lie in preventing signal deterioration over time rather than immediately boosting initial signal amplitude [5].

These results argue for a rational pairing of CAR designs with metabolic interventions based on tumor context. In solid tumors where antigen expression is low or heterogeneous and the microenvironment is rich in suppressive metabolites, a metabolically refueled CAR (or a CAR with co-stimulation that strongly activates NF-κB, or both) could markedly improve efficacy. For example, HER2-positive cancers often have variable antigen density; using MR might raise CAR T activity from a borderline level to one sufficient for tumor control. In contrast, for tumors with uniformly high antigen density (e.g., GPC3 in HCC) where CAR T cells will be highly activated anyway, MR may primarily function to sustain T cell fitness (through adenosine removal and inosine supply) during repetitive engagements, rather than visibly altering early transcriptomic signatures. Either way, monitoring NF-κB-linked pathways and TF signatures in patient CAR T cells could provide biomarkers of response to MR or similar interventions. For instance, an increase in NF-κB/AP-1 target gene expression or in nuclear p65 localization in patient T cells post-infusion could indicate effective metabolic refueling or co-stimulation enhancement, whereas lack of such changes might suggest the need for adjunct therapies.

In addition, while our study focused on early, antigen-independent transcriptional changes, it sets the stage for more complex evaluations under tumor conditions. Applying a relaxed significance threshold, such as an adjusted *p*-value of 0.05 and a log2 fold change cut off of 0, increases the risk of including genes with minimal or biologically insignificant changes in expression. While this approach can identify a larger number of differentially expressed genes, it may also yield results that are more prone to false positives and may include many changes that are statistically but not biologically meaningful, potentially complicating interpretation and validation. Important next steps include incorporating serial antigen stimulation or tumor co-culture to simulate chronic exposure, and single-cell analyses to resolve differentiation trajectories. Our results predict that MR-CAR T cells will better resist exhaustion under repetitive stimulation—an idea supported by the maintained NF-κB activity and higher c-Jun/Fos (AP-1) levels, since sustained AP-1 has been linked to exhaustion prevention [32]. Future work can directly measure NF-κB dynamics (e.g., real-time p65 nuclear translocation or IκBα degradation assays) in CAR T cells with and without metabolic support, to confirm that MR prolongs the NF-κB signaling pulses that drive effective responses. By keeping NF-κB within an optimal range—not too low (exhausted) and not aberrantly high (hyperactivated)—metabolic refueling and other engineering tweaks may maximize CAR T cell durability and functionality in solid tumors. Our data provide a framework for such “Goldilocks” tuning of CAR T signaling, highlighting NF-κB/AP-1 as both a readout and a control point for next-generation CAR design.

Finally, safety considerations are important for metabolic refueling strategies. Since extracellular adenosine contributes to immunoregulation [34], engineered adenosine degradation may raise inflammatory tone and cytokine programs and potentially influence systemic inflammation or cytokine-associated toxicities. On the other hand, upon metabolic reprogramming, CAR T cells might be less susceptible to stress and have less inflammatory behavior. Since the present work does not evaluate toxicity, future work should monitor safety (e.g., serum cytokines, weight loss, and histopathology) and controllability mechanisms (e.g., dose optimization) to balance enhanced function with inflammatory risk.

## 5. Conclusions

This study demonstrates that CAR T cell architecture, including co-stimulatory domain selection and metabolic enhancements, shapes transcriptional state even in the absence of antigen stimulation. Tonic signaling, particularly through NF-κB and related inflammatory regulators, emerges as a consistent feature of CAR expression. However, this baseline activity differs substantially between CD28- and 4-1BB–containing constructs. Metabolic refueling via ADA/CD26 further amplifies these programs. Together, these results suggest that basal CAR signaling is a tunable property that influences T cell preparedness and potential fitness. Rational engineering that balances tonic signaling, co-stimulatory signals, and metabolic support may be key to producing CAR T cells with optimized persistence, function, and adaptability in the solid tumor setting.

### Limitations

Even though our study provides important insights into the transcriptomic landscape of CAR T cell architecture, it also has some limitations. First, between model comparisons (GPC3 vs. HER2) are partially confounded by antigen-binding domain properties, CAR expression density [31], in addition to co-stimulatory domain choice; accordingly, we interpret these differences as model-to-model rather than CD28 vs. 4-1BB effects. Second, NF-κB engagement and tonic signaling are inferred indirectly from pathway enrichment and transcription factor activity inference; we did not measure NF-κB signaling dynamics directly. Third, we used a discovery-oriented differential expression approach and used modest fold change and adjusted *p* value cutoffs. Identification of signatures that remain robust under stricter criteria will be crucial. In addition, further experimentation is required to understand how these detected transcriptional changes impact the functionality of CAR T cells. Finally, our study does not address transcriptional changes that occur upon antigen stimulation. Future studies will investigate the changes that occur after antigen stimulation on CAR T cells.

## Data Availability

The data can be found in Gene Expression Omnibus (GEO) under the accession number GSE262447.

## Supplementary Materials

The following supporting information can be downloaded at: https://www.mdpi.com/article/10.3390/metabo16010052/s1, Figure S1: Number of differentially expressed genes between NT controls and CAR T cells. Figure S2: CAR and MR-CAR share core, conserved, inflammatory Hallmark pathways. Figure S3: GPC3-MR vs. GPC3-CAR GO enrichment details. Figure S4: HER2-MR vs. HER2-CAR GO enrichment details. Figure S5: KEGG analyses reveal reinforced cytokine/NF-κB networks with metabolic refueling. Table S1: List of differentially expressed genes across all samples_related to S1c. Table S2: DoRothEA results_related to Figure 4.

## Abbreviations

The following abbreviations are used in this manuscript:

CAR	Chimeric antigen receptor
NF-κB	Nuclear factor kappa-B
HER2	Human epidermal growth factor receptor
GPC3	Glypican 3
ADA1	Adenosine deaminase 1
MR	Metabolic refueling
GO	Gene ontology
KEGG	Kyoto Encyclopedia of Genes and Genomes
