# Supplementary material for: CAR Intrinsic Design Pre-Shapes Transcriptional and Metabolic Networks in CAR T Cells
Source: Metabolites. 2026 Jan 7;16(1):52. doi: 10.3390/metabo16010052 (PMC12844403; doi:10.3390/metabo16010052)
Supplement: Supplementary file 1 [file metabolites-16-00052-s001.zip › Suppl Figures.pdf]

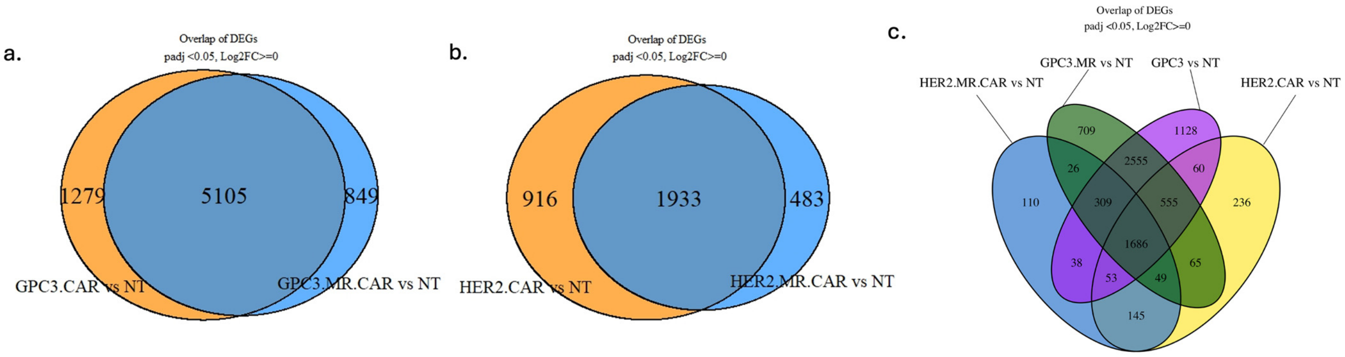

Figure S1. Number of differentially expressed genes between NT controls and CAR T cells. Venn diagrams display overlaps of all DEGs (padj < 0.05, log2FC >= 0) for GPC3 CAR T vs. NT and GPC3 MR CAR T vs. NT (**a**) and for HER2 CAR T vs. NT and HER2 MR CAR T vs. NT (**b**), and between all samples (**c**). Numbers indicate set sizes and intersections. Large overlaps in both antigens define a shared activation core across CAR constructs; however, the size of the DEG sets and the overlap fraction are greater in GPC3 than in HER2. DEGs reflect genes that changed either direction.

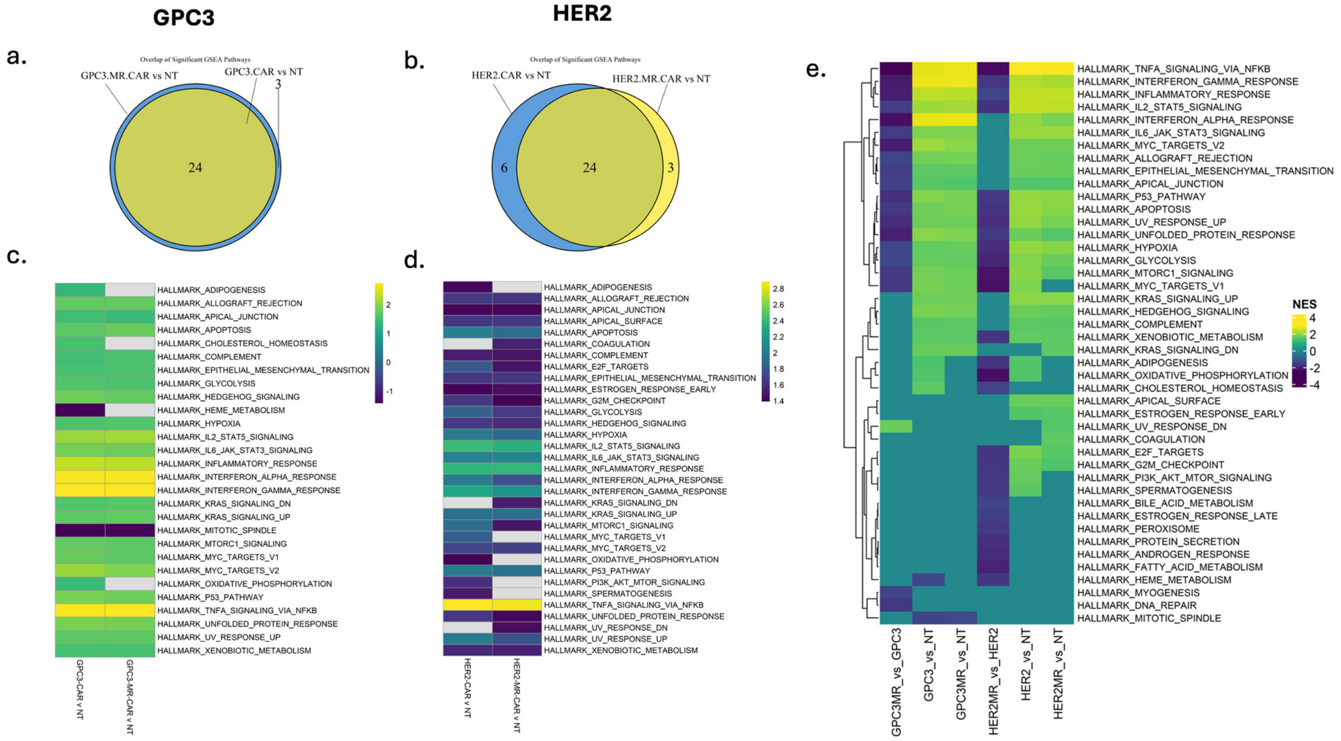

Figure S2. CAR and MR-CAR share core, conserved, inflammatory Hallmark pathways. (a–b) Venn diagrams showing the overlap of significantly enriched Hallmark gene sets for CAR and MR-CAR cells in comparison to NT in GPC3 (**a**) and HER2 (**b**). Numbers denote shared and unique sets. (**c–d**) Heatmaps of normalized enrichment scores (NES) for significant Hallmarks in GPC3 (**c**) and HER2 (**d**), comparing CAR vs. NT and MR-CAR vs. NT; red = positive enrichment, blue = negative. (**e**) Clustered heatmap of NES across all indicated comparisons (GPC3 CAR T vs. NT, GPC3 MR CAR T vs. NT, HER2 CAR T vs. NT, HER2 MR CAR T vs. NT, GPC3 MR CAR T vs. GPC3 CAR T, HER2 MR CAR T vs. HER2 CAR), showing concordant positive enrichment of inflammatory modules (e.g., TNF-α via NF-κB, interferons, JAK–STAT) and MR-associated amplification, with larger effects in GPC3.

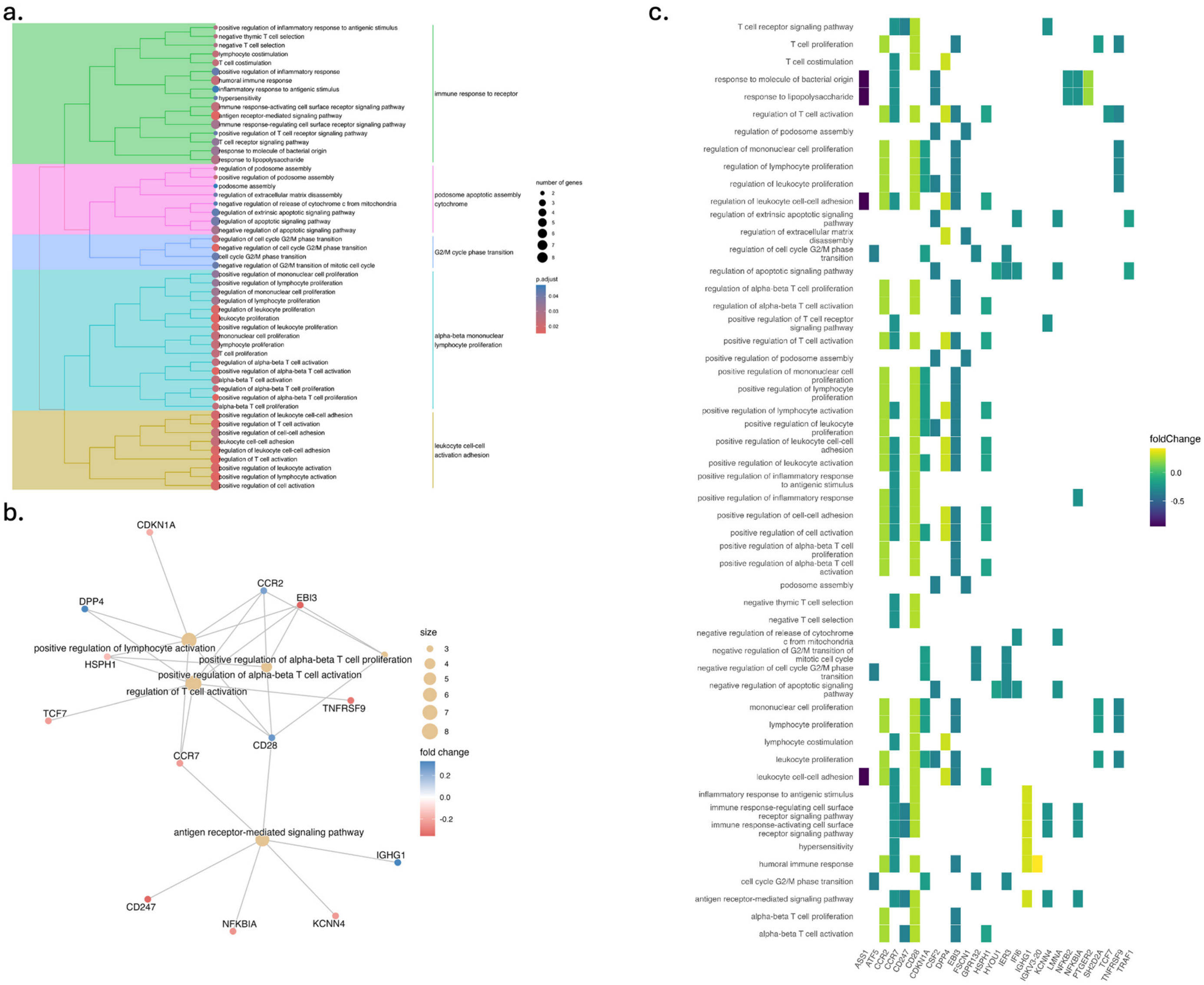

Figure S3. GPC3-MR vs. GPC3-CAR GO enrichment details. (**a**) Dot plot of GO Biological Process terms showing the top 20 pathways (dot size = gene count; color = adjusted *p*). The accompanying dendrogram visualizes semantic similarity and functional clustering of *all* GO terms identified using *p* value cut-offs. (**b**) Cnet network depicting genes associated with the top 5 enriched GO pathways; node color encodes log2 fold-change (Log2FC). (**c**) Heatmap of log2FC for genes mapped to all enriched GO pathways.

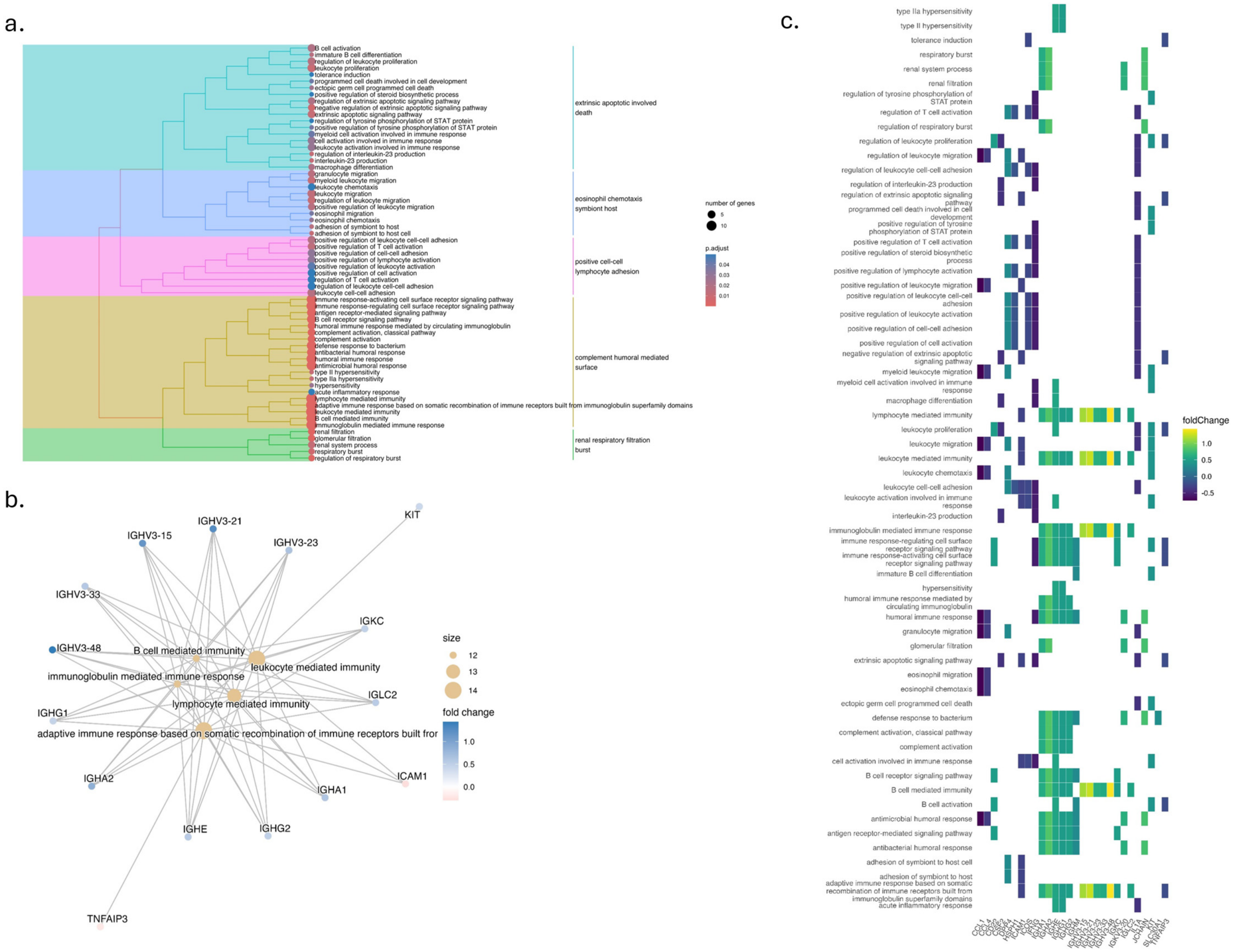

Figure S4. HER2-MR vs. HER2-CAR GO enrichment details. (a) Dot plot of GO Biological Process terms showing the top 20 pathways (dot size = gene count; color = adjusted *p*). The accompanying dendrogram visualizes semantic similarity and functional clustering of *all* GO terms identified using *p* value cut-offs. (b) Cnet network depicting genes associated with the top 5 enriched GO pathways; node color encodes log2 fold-change (Log2FC). (c) Heatmap of log2FC for genes mapped to all enriched GO pathways.

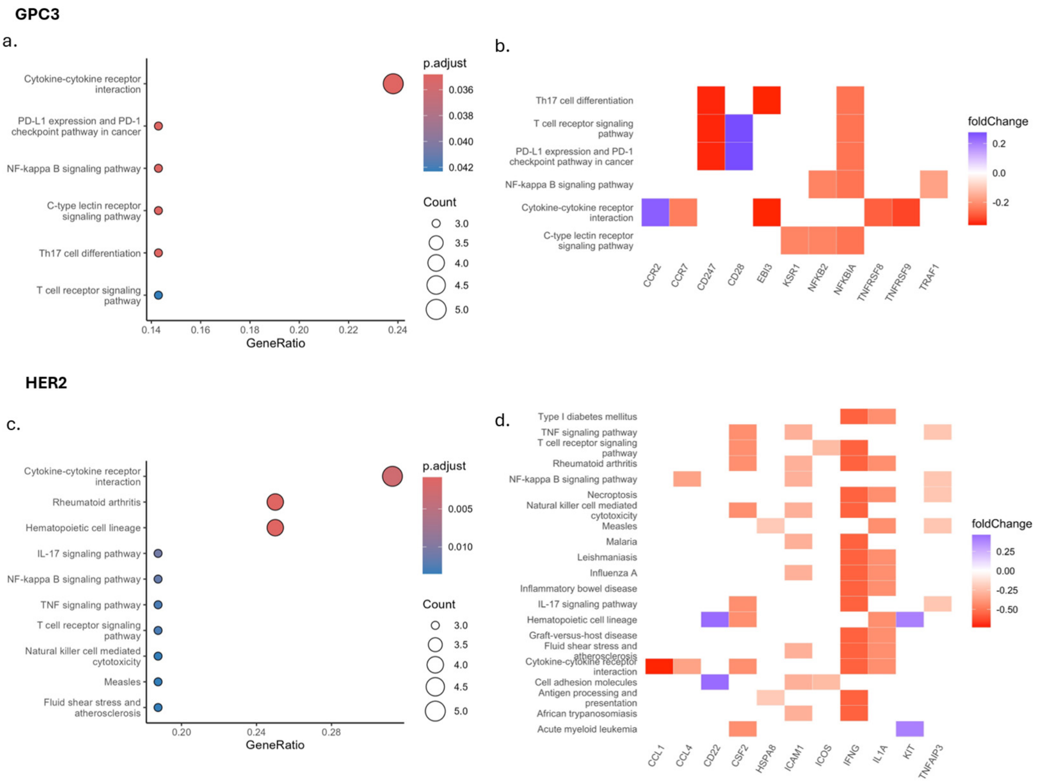

Figure S5. KEGG analyses reveal reinforced cytokine/NF-κB networks with metabolic refueling. (a and c) KEGG dotplot of significantly enriched pathways (node color = adjusted *p*; x-axis = GeneRatio; bubble size = mapped gene count) for MR CAR T vs. CAR T (GPC3 (**a**), HER2 (**c**). (b and d) Gene–pathway heatmap showing log2FC for representative genes mapped to the enriched KEGG terms in GPC3 (**b**) and HER2 (**d**)
